# Composition of the Essential Oil of *Salvia ballotiflora* (Lamiaceae) and Its Insecticidal Activity

**DOI:** 10.3390/molecules20058048

**Published:** 2015-05-05

**Authors:** Norma Cecilia Cárdenas-Ortega, Marco Martín González-Chávez, Rodolfo Figueroa-Brito, Antonio Flores-Macías, Diana Romo-Asunción, Diana Elizabeth Martínez-González, Víctor Pérez-Moreno, Miguel Angel Ramos-López

**Affiliations:** 1 Facultad de Ciencias Químicas, Universidad Autónoma de San Luis Potosí, Av. Dr. Manuel Nava 6, Zona Universitaria, C.P. 78290 San Luis Potosí, S.L.P., Mexico; 2 Centro de Desarrollo de Productos Bióticos, Instituto Politécnico Nacional, Carretera Yautepec-Jojutla, km 6, Calle Ceprobi No. 6, Col. San Isidro, C.P. 62731 Yautepec, Morelos, Mexico; 3 Departamento de Producción Agrícola y Animal, Universidad Autónoma Metropolitana Unidad Xochimilco, Calzada del Hueso 1100, Col. Villa Quietud, C.P. 04960 Deleg. Coyoacán, D.F., Mexico; 4 Estudiante de la Maestría en Ciencias Agropecuarias, Universidad Autónoma Metropolitana Unidad Xochimilco, Calzada del Hueso 1100, Col. Villa Quietud, C.P. 04960 Deleg. Coyoacán, D.F., Mexico; 5 Facultad de Química, Universidad Autónoma de Querétaro, Cerro de las Campanas s/n, Col. Las Campanas, C.P. 76010 Santiago de Querétaro, Querétaro, Mexico

**Keywords:** *Spodoptera frugiperda*, essential oil, β-caryophyllene, caryophyllene oxide

## Abstract

Essential oils can be used as an alternative to using synthetic insecticides for pest management. Therefore, the insectistatic and insecticidal activities of the essential oil of aerial parts of *Salvia ballotiflora* (Lamiaceae) were tested against the fall armyworm *Spodoptera frugiperda* (Lepidoptera: Noctuidae). The results demonstrated insecticidal and insectistatical activities against this insect pest with concentrations at 80 µg·mL^−1^ resulting in 20% larval viability and 10% pupal viability. The larval viability fifty (LV_50_) corresponded to a concentration of 128.8 µg·mL^−1^. This oil also increased the duration of the larval phase by 5.5 days and reduced the pupal weight by 29.2% withrespect to the control. The GC-MS analysis of the essential oil of *S. ballotiflora* showed its main components to be caryophyllene oxide (15.97%), and β-caryophyllene (12.74%), which showed insecticidal and insectistatical activities against *S. frugiperda.* The insecticidal activity of β-caryophyllene began at 80 µg·mL^−1^, giving a larval viability of 25% and viability pupal of 20%. The insectistatic activity also started at 80 µg·mL^−1^ reducing the pupal weight by 22.1% with respect to control. Caryophyllene oxide showed insecticidal activity at 80 µg·mL^−1^ giving a larval viability of 35% and viability pupal of 20%.The insectistatic activity started at 400 µg·mL^−1^ and increased the larval phase by 8.8% days with respect to control. The LV_50_ values for these compounds were 153.1 and 146.5 µg·mL^−1^, respectively.

## 1. Introduction

The caterpillar of *Spodoptera frugiperda* (Lepidoptera: Noctuidae) is a significant polyphagous insect pest of agricultural importance, not only for the damage it causes, but also due to its control difficulties [1]. This species inhabits the American continent from southern Canada to Argentina and causes considerable economic losses in several important crops such as maize, sorghum, rice, cotton, alfalfa, forage grasses, and occasionally other crops in the majority of the countries within its range [2,3,4]. The principal method to control this insect is the application of synthetic insecticides which yield effective results over a short time period, however, resistance development to these insecticides is also very fast, leading farmers to increase dosages or change the active ingredient frequently [5,6,7].

The implementation of Integrated Pest Management Programs appear to be a suitable alternative to managing noctuid insects by combining different methods that include the use of botanical extracts as insecticides [8]. Due to this, there has been a growing interest in botanical resources with activity against insect pests. This need has originated from the demand to provide alternatives to reduce the use of synthetic insecticides, which can have adverse effects on the environment [9]. The use of essential oils against insect pests has been frequently proposed. According Koul *et al.* [10] these substances are defined as any volatile oil(s) that have strong aromatic components and that give a distinctive odor, flavor or scent to a plant. These oils can be found in glandular hairs or secretory cavities of plant-cell walls and are present as droplets of fluid in the leaves, stems, bark, flowers, roots, and/or fruits of different plants. The essential oils provide various functions for the plants including: (i) attracting or repelling insects; (ii) protecting them from heat or cold; and (iii) utilizing chemical constituents in the oil as means of defense. Many plant essential oils obtained from *Salvia* species and their constituent compounds have been evaluated against insect pests and a number of them have shown considerable promise for the development of natural repellents/insecticides [11,12,13,14]. The *Salvia* genus is the most diverse of Lamiaceae Family, with over 1000 species around the world distributed in tropical and subtropical zones. In Mexico, there are at least 300 species reported [15]. This genus demonstrates an affinity to pine and oak forests as well as cloud and tropical deciduous forest, but also has shown remarkable diversity and endemism in arid and desert zones. The Mexican states richest in these species are: Oaxaca, Guerrero, Puebla, Jalisco, Michoacán, Coahuila, Baja California Sur, Tamaulipas and San Luis Potosí [16].

Therefore, the aim of this study was to determine insecticidal and insectistatic activities against *S. frugiperda* of the essential oil of the aerial parts of *Salvia ballotiflora* (Lamiaceae) and its main components.

## 2. Results and Discussion

**Figure 1:**
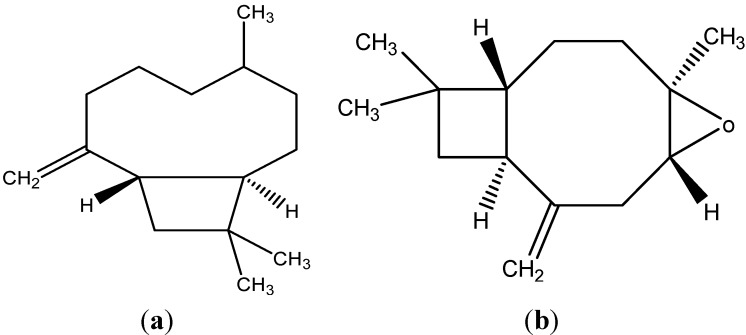
Structures of β-caryophyllene (**a**) and caryophyllene oxide (**b**).

The GC-MS analysis of the aerial parts *S. ballotiflora* essential oil, showed 37 different compounds (Table 1), corresponding to 67% of the total composition. The main components were sesquiterpenes such as β-caryophyllene (C_15_ H_24_) with 12.74% and caryophyllene oxide (C_15_ H_24_ O) with 15.97% (Figure 1). In other essential oils of *Salvia* species, the main components were similar, with *Salvia verticillata* containing 16.03% and 15.24% of β-caryophyllene and caryophyllene oxide, respectively [17]; for *Salvia hydrangea*the composition was 25.1% and 11.5% [18]. In another study of the essential oils from *S. verticillata*, *Salvia sclarea*, *Salvia chloroleuca*, and *Salvia multicaulis*, β-caryophyllene was the principal compound in each of the species with 31.5%, 9%, 9% and 8.9%, respectively [19]. Essential oil of *Salvia aethiopi* and *Salvia nemorosa* also contained high concentrations of β-caryophyllene (24.8% and 19.03% for each plant) [17]. In the essential oil from *Salvia verbenaca*, caryophyllene oxide was the main component with 7.28% [20]. According Liu *et al.* For *Salvia umbratica*, had a caryophyllene oxide concentration of 8.42% [12]. On the other hand, Lima *et al.* [21] found (*E*)-caryophyllene (15.35%), α-eudesmol (14.06%), β-eudesmol (8.74%) and γ-eudesmol (7.64%) as a principal components of essential oil of aerial parts of *Salvia microphylla*.

**Table 1:** *S. ballotiflora* Essential Oil Chemical Composition.

No.	RT	Component	KIL	KI	*Peak Area%
1	9.08	(−)-β-Pinene	961.7	950	0.49
2	10.15	3-Octanol	985	995	0.61
3	11.51	Eucalyptol	1023	1027	1.16
4	12.42	β-*cis*-Ocimene	1024	1046	0.44
5	14.8	Linalol	1081	1096	0.9
6	15.31	*exo*-Fenchol	1112	1107	0.12
7	15.67	Isophorone	1094	1115	0.3
8	16.67	(−)-Alcanfor	1146	1137	0.16
9	17.71	Camphol	1148	1159	0.46
10	18.28	Terpinen-4-ol	1161	1171	0.42
11	18.94	α-Terpineol	1172	1186	0.95
12	19.77	*exo*-2-Hydroxycineole	1212	1204	0.55
13	23.28	Bornylacetate	1269	1282	0.47
14	25.54	δ-EIemene	1334	1333	0.43
15	26.52	Eugenol	1337	1355	0.66
16	26.96	Ylangene	1392	1365	0.14
17	27.81	β-Cubebene	1384	1384	0.17
18	27.9	β-Elemene	1387	1386	0.44
19	29.04	β-Caryophyllene	1424	1413	12.74
20	29.75	α-Bergamotene	1407	1431	0.51
21	30.41	Humulene	1456	1447	3.87
22	30.79	(+)-*epi*-Bicyclosesquiphellandrene	1435	1456	0.22
23	31.41	γ-Muurolene	1494	1472	1.62
24	31.73	α-Curcumene	1472	1480	0.56
25	32.13	Eremophilene	1486	1490	0.84
26	32.37	α-Muurolene	1490	1496	0.66
27	32.91	γ-Cadinene	1505	1509	1.21
28	33.32	δ-Cadinene	1514	1519	3.98
29	34.04	α-Calacorene	1539	1537	0.16
30	35.01	Nerolidol	1545	1561	1.3
31	35.65	Caryophylene oxide	1576	1577	15.97
32	35.92	α-Acorenol	1598	1584	0.93
33	36.59	Longifolenaldehyde	1581	1601	2.24
34	36.74	2-Methylene-6,8,8-trimethyltricyclo [5.2.2.0 (1,6)] undecan-3-ol	1599	1605	1.07
35	37.87	τ-Cadinol	1628	1636	2.35
36	38.34	α-Cadinol	1641	1649	3.27
37	51.74	Abietatriene	2039	2062	0.11

Retention time (in minutes). KI Kovats index, relative to C_6_-C_26_ *n*-alkanes on the HP-5MS column. KIL Kovats index on an apolar column; * Values reported as a percentage of the total area.

The insecticidal activity of *S. ballotiflora* essential oil at 1000, 600, 400, 120 and 80 µg·mL^−1^ showed larval viability of 0%, 5%, 10%, 10% and 20%, respectively. The pupal viability at 600, 400, 120 and 80 µg·mL^−1^ was 5%, 10%, 10% and 20%, respectively. The LV_50_ was 128.8 µg·mL^−1^ (Table 2). The insectistatic activity increased the larval duration by 30.5, 8.0, 5.5, and 5.5 days at 600, 400, 120, 80 µg·mL^−1^ with respect to the control. Regarding the pupal duration there was an increase of 1.6 days at 400 µg·mL^−1^. Moreover the pupal weight was reduced by 52%, 39%, 29%, 29% at 600, 400, 120, 80 µg·mL^−1^, respectively, when compared to the control pupal weight. On the other hand, Hosseini *et al.* [22] showed that the CL_50_ of essential oil of *Salvia leriifolia* against adults of *Sitophilus granarius* (Coleoptera: Curculionidae) and *Rhyzopertha dominica* (Coleoptera: Bostrichidae), after 24 h were 79.17 μL·L^−1^ and 25.87 μL·L^−1^ respectively. Moreover, Khiyari *et al.* [23] also reported the insecticidal activity of two essential oils of *Salvia aucheri*, one from a wild plant and another from a cultivated plant against adults of *Tribolium castaneum* (Coleoptera: Tenebrionidae); the authors determined the CL_50_ and CL_90_ for each, and the wild plant the values were 1.00 μL·cm^−2^, 1.72 μL·cm^−2^, respectively, and for the cultivated plant the values were 1.25 μL·cm^−2^ and 2.03 μL·cm^−2^. Ulukanli *et al.* [14] evaluated the activity of essential oil of *Salvia tomentosa* against adults of *Acanthoscelides obtectus* (Coleoptera: Bruchidae) and *T. castaneum*; this oil caused 100% mortality at 50 µL·L^−1^ air and 200 µL·L^−1^ air for each insect. Liu *et al.* [12]. They also reported a LD_50_ of 18.12 µg·adult^−1^ of the essential oil of *S. umbratica* against *Sitophilus zeamais* (Coleoptera: Curculionidae). The essential oils from *Salvia hydrangea*, *Salvia numerosa*, *Salvia multicaulis* and *Salvia sclarea* (Lamiaceae) were also tested against adults of *S. granarius*. These essential oils caused 67.33%, 39.73%, 55.21% and 41.76% mortality at 153.84 µL·L^−1^ air concentration [24]. On the other hand, the essential oils of *Salvia limbata* and *Salvia nemorosa* (Lamiaceae) resulted in average mortality rates against *S. granarius* (10% and 14%, respectively) at 76.91 µL·L^−1^ air concentration. The mortality rate increased with the concentration of the essential oils and the length of the exposure period [25].

**Table 2:** Insecticide and insectistatic activities of *S. ballotiflora* essential oil against *S. frugiperda*.

Concentration (µg·mL^−1^)	Viability (%)		Duration (d)		Pupal Weight (mg)
	Larva	Pupa	Larva	Pupa	
1000	-	-	-	-	-
600	5.0 ± ND *	-	57.0 ± ND *	-	106.0 ± ND *
400	10 ± 6.8 *	5.0 ± ND *	34.5 ± 2.5 *	12.0 ± ND *	133.5 ± 9.5 *
160	10 ± 6.8 *	10 ± 6.8 *	32.0 ± 1.0 *	11.0 ± 1.0	155.5 ± 9.5 *
80	20 ± 9.2 *	10 ± 6.8 *	32.0 ±2.8 *	10.5 ± 0.5	156.5 ± 3.9 *
0	95 ± 5.0	90 ± 6.8	26.5 ± 0.5	10.2 ± 0.4	221.1 ± 9.1
LV_50_	0.1288 × 103 (0.0835–0.1590) µg·mL^−1^				

Results are the mean of at least 20 determinations ± standard error. * Significantly different from control *p* < 0.05. LV_50_ was calculated using larval viability, in parentheses confidence intervals *p* < 0.05.

Others studies have reported insectistatic activity with some *Salvia* species, Karahroodi *et al.* [26] showed 56% and 32% repellency with the essential oils of *Salvia multicaulis* and *Salvia officinalis* (Lamiaceae), respectively, against female adults of *Plodia interpunctella* (Lepidoptera: Pyralidae) at 15.39 µL·L^−1^ air concentration using an olfactometer. Additionally, Lakshmanan *et al.* [27] reported antifeedant activity rates of 85.56% on *Spodoptera litura* (Lepidoptera: Noctuidae), 45.64% on *Helicoverpa armigera* (Lepidoptera: Noctuidae) and 79.45% on *Achaea janata* (Lepidoptera: Noctuidae) with 1000 µg·mL^−1^ of *S. officinalis* essential oil*.* Conti *et al.* [28] described 100% repellency activity within the first 15 minutes at 0.04, 0.2 and 0.4 μL·cm^−2^ with the essential oils from *Salvia dorisiana*, *Salvia longifolia*, *Salvia sclarea* against adults of *Aedes albopictus* (Diptera: Culicidae), and the repellency was 90% after 90 minutes of exposure with 0.4 μL·cm^−2^ from *S. dorisiana*.

Specifically, chloroform extracts from the aerial parts of four *Salvia* species (Lamiaceae) were tested for insectistatic and insecticidal activities against *S. frugiperda*. All extracts showed both activities. Extracts from *Salvia keerlii*, and *Salvia ballotiflora*, had moderate insecticidal activity (LV_50_ 1527 and 1685 µg·mL^−1^, respectively), and the *S. ballotiflora* extract increased the larval and pupal phases by 5.2 and 2.9 days, respectively, and reduced the pupal weight by 13.2% [29]. In this study, essential oil from aerial parts of *S. ballotiflora* had high insecticidal activity (larval viability of 0%, 5%, 10%, 10% and 20%, a 1000, 600, 400, 120 and 80 µg·mL^−1^, respectively). The insectistatic activity increased the larval duration by 30.5, 8.0, 5.5, and 5.5 days, and reduced the pupal weight by 29% a 52% at 600, 400, 120, 80 µg·mL^−1^ respectively in regards to the control.

The insecticide activity of β-caryophyllene at 1000, 600, 400, 160 and 80 µg·mL^−1^ showed larval viability of 5%, 20%, 20%, 25% and 25% respectively and the pupal viability was 0%, 5%, 5%, 20% and 20% respectively. The LV_50_ was 153.1 µg·mL^−1^ (Table 3). The insectistatic activity caused the larval duration increases of 24.8, 11.8, 9.8, and 5.8 days at 1000, 600, 400 and 160 µg·mL^−1^ with respect to the control, and at all concentrations there was no adult emergence, therefore is no data for pupal duration. Moreover, the pupal weight was reduced by 52%, 39%, 29%, 29% at 600, 400, 160, 80 µg·mL^−1^ respectively in regards to the control pupal weight. The insecticidal activity of caryophyllene oxide at 1000, 600, 400, 160 and 80 µg·mL^−1^ showed larval viability of 0%, 5%, 5%, 20% and 35%, respectively, and the pupal viability at same concentrations was 0%, 5%, 5%, 20% and 20%, respectively. The LV_50_ was 146.5 µg·mL^−1^ (Table 4). The insectistatic activity began to increase the larval duration by 9.8, and 8.8 days at 600 and 400 µg·mL^−1^ with respect to the control and at all concentrations there was no adult emergence, therefore there is no data for pupal duration. Moreover the pupal weight was reduced by 16.7% at 600 µg·mL^−1^ in respect to the control pupal weight.

**Table 3:** Insecticide and insectistatic activities of β-caryophyllene against *S. frugiperda*.

Concentration (µg·mL^−1^)	Viability (%)		Duration (d)		Pupal Weight (mg)
	Larva	Pupa	Larva	Pupa	
1000	5.0 ± 5.0 *	-	50.0 ± ND *	-	116 ± ND *
600	20.0 ± 9.2 *	5.0 ± 5.0 *	37.0 ± ND *	-	109.2 ± 34.6 *
400	20.0 ± 9.2 *	5.0 ± 5.0 *	35.0 ± ND *	-	147 ± 49.0 *
160	25.0 ± 9.9 *	20.0 ± 9.2 *	31.0 ± 1.0 *	-	155.5 ± 9.5 *
80	25.0 ± 9.9 *	20.0 ± 9.2 *	27.6 ± 2.2	-	163.7 ± 16.7 *
0	95.0 ± 5.0	90.0 ± 6.9	25.2 ± 1.4	9.2 ± 0.8	210.1 ± 14.0
LV_50_	0.1531 × 103 (0.1045–0.1842) µg·mL^−1^				

Results are the mean of at least 20 determinations ± standard error. * Significantly different from control *p* < 0.05. LV_50_ was calculated using larval viability, in parentheses confidence intervals *p* < 0.05.

Liu *et al.* [30], reported the insecticidal activity and fumigant toxicity of caryophyllene oxide on two insect pests. These results demonstrated a LD_50_ of 34.09 mg·adult^−1^ and LC_50_ of 17.02 mg·L^−1^ against *S. zeamais*, and a LD_50_ of 45.56 mg·adult^−1^ and LC_50_ of 15.98 mg·L^−1^ against *T. castaneum*. This sesquiterpene was reported as one of the main components (9.32%) of the essential oil of fruits of *Illicium pachyphyllum* (Schisandraceae). The essential oil of the aerial parts of *Saussurea nivea* (Asteraceae) used as a fumigant LC_50_ of 8.89 mg·L^−1^ and contact LC_50_ of 10.56 µg·adult^−1^ against *S. zeamais*. The main components of this oil were (+)-limonene (15.46%), caryophyllene oxide (7.62%), linalool (7.20%), α-pinene (6.43%), β-pinene (5.66%) and spathulenol (5.02%) [31]. Tchoumbougnang *et al.* [32], showed that essential oils of fruits of *Piper capense* (LD_50_ 26.4 µL·g^−1^), *Piper guineese* (LD_50_ 16.1 µL·g^−1^) and *Piper nigrum* (LD_50_ 10 µL·g^−1^) had insecticidal activity against *S. zeamais*, and that in those oils β-caryophyllene was a compound with 3.4%, 20.8% and 12.8% in each of the species.

**Table 4:** Insecticide and insectistatic activities of Caryophyllene oxide against *S. frugiperda*.

Concentration (µg·mL^−1^)	Viability (%)		Duration (d)		Pupal Weight (mg)
	Larva	Pupa	Larva	Pupa	
1000	-	-	-	-	-
600	5.0 ± 5.0 *	5.0 ± 5.0 *	35.0 ± ND *	-	175.0 ± ND *
400	5.0 ± 5.0 *	5.0 ± 5.0 *	34.0 ± ND *	-	195.0 ± 9.5
160	20.0 ± 9.2 *	20.0 ± 9.2 *	28.3 ± 1.5	-	200.8 ± 8.9
80	35.0 ± 3.7 *	20.0 ± 9.2 *	26.3 ± 1.9	-	201.3 ± 9.1
0	95.0 ± 5.0	90.0 ± 6.88	25.2 ± 1.4	9.2 ± 0.8	210.1 ± 14.0
LV_50_	0.1465 × 103 (0.1036–0.1742) µg·mL^−1^				

Results are the mean of at least 20 determinations ± standard error. * Significantly different from control *p* < 0.05. LV_50_ was calculated using larval viability, in parentheses confidence intervals *p* < 0.05.

Chaubey [33], evaluated the essential oil of *Zingiber officinale* (Zingiberaceae) and *Piper cubeba* (Piperaceae) and the main compounds of those oils, that worked against adults and larvae of *Tribolium castaneum* (Coleoptera: Tenebrionidae) and adults of *Sitophilus oryzae* (Coleoptera: Curculionidae) were β-caryophyllene and α-pinene. β-caryophyllene showed LD_50_ values of 0.173 µL cm^−2^ after 24 h against *T. castaneum* adults, 0.17 µL cm^−2^ after 24 h against *T. castaneum* larvae, and 0.159 µL cm^−2^ after 24 h against *S. oryzae* adults. Benelli *et al*. [34] tested the essential oil of *Hyptis suaveolens* (Lamiaceae) and its principal compounds against *Sitophilus granarius* (Coleoptera: Curculionidae), β-caryophyllene (11.2%) showed 65% of repellence activity.

## 3. Experimental Section

### 3.1. Plant Material

The aerial parts (leaves, stems and flowers) of *S. ballotiflora* were collected in the Municipio of Guadalcázar, San Luis Potosí, México, at 1640 m.a.l.s., in September of 2013, the Taxonomic authentication was performed by José García-Pérez at the Isidro Palacios Herbarium of the Universidad Autónoma de San Luis Potosí. A voucher specimen was stored (SLPM 43013).

### 3.2. Essential Oil Extraction

Aerial parts of the plant weighing approximately 2 kg, were submitted to hydrodistillation for 3 hours. The mixture obtained was treated with ethyl ether, then the organic phase was separated and concentrated with a rotatory evaporator at 18 °C. The essential oil obtained was dehydrated with anhydrous sodium sulphate, and the ethyl ether residue was eliminated under vacuum, to give a yellow amber essential oil with a density of 0.6836 g·mL^−1^ at 20 °C, and refraction index of 1.4095 at 25 °C. The yield was (0.47 *w*/*w*). The oil was protected from direct light and stored at 4 °C until its use.

### 3.3. Chemicals

β-caryophyllene and caryophyllene oxide standards were purchased from Sigma-Aldrich (St. Louis, MO, USA).

### 3.4. Identification of Essential Oil Main Components

*S. ballotiflora* essential oil samples (20 µL) were diluted with acetone (1 mL). The essential oil was analyzed on an Agilent Technologies (Santa Clara, CA, USA) 6890N GC equipped with an HP-5MS column (30 m in length; 25 mm internal diameter; 0.25 μm film thicknesses) and an Agilent EM 5973 detector, at 250 °C. The carrier gas was helium, with a flow rate of 1 mL·min^−1^; the split ratio was 2:1. The column temperature was initially 50 °C (for 3 min) and was gradually increased to 240 °C, at 3 °C·min^−1^; this temperature was held for 2 min. The injector temperature was 250 °C and 1 μL of essential oil was injected as a duplicate. The spectra were collected at 71 eV ionization voltages and the analyzed mass range was 15–600 *m*/*z*. The identification of the components were confirmed by comparison of the retention indices with those of authentic compounds using the Kovats index, based on n-alcanes C6-C26 with the Wiley09/NIST11 library. The identification of the two main components was confirmed by comparison of the retention indices to those of authentic compounds.

### 3.5. Insect Rearing

Fall armyworm (*S. frugiperda*) larvae were reared in the Insecticide Natural Compounds Laboratory from the Chemistry Faculty of the Autonomous University of Querétaro, according to the Bergvinson and Kumar [35] methodology using the following parameters: temperature 25 ± 2 °C with a relative humidity of 70% and 12/12 h light/dark cycles. For 1 kg diet feed for *S. frugiperda*, the following ingredients were used: 800 mL of distilled water, 60 g diet (Product# F0635 S.W. Corn Borer, Bio-Serv, Frenchtown, NJ, USA), 20 g sterile corn spike, 100 g ground corn, 40 g brewer’s yeast, 10 g vitamins (vitamin mix fortification lepidoptera, Bio-Serv), 10 g agar, 1.7 g sorbic acid (dissolved in the ethanol), 17 mL ethanol, 2.5 mL formaldehyde, 1.7 g methyl *p*-hydroxybenzoate and 0.6 g neomycin sulfate.

### 3.6. Bioassay

For the bioassay, first instar larvae of *S. frugiperda* were used. Groups of 20 larvae were randomly selected for each concentration of *S. ballotiflora* essential oil, β-caryophyllene and caryophyllene oxide. Preliminary screening of essential oil for each compound was carried out at five concentrations (0.1, 1, 10, 100 and 1000 µg·mL^−1^). Based on the preliminary screening results, the concentration-dependent levels were selected (80, 160, 400, 600 and 1000 µg·mL^−1^). The test included a negative control (diet only). The essential oil, β-caryophyllene and caryophyllene oxide were mixed with the larvae diet ingredients during preparation without use of any solvent, according the methodology of Ramos-López *et al.* [36]. The effect of essential oil and the compounds were monitored during all larval stages “known as the larval-phase duration”, and the pupal stage “called the pupal-phase duration”. The number of pupae formed (larval viability), number of adults formed (pupal viability), and weight of pupae at 24 hours were assessed. The larval viability (LV_50_) corresponded to 50% of the larvae of fall armyworm during all larval phases for each extract.

### 3.7. Statistical Analysis

Statistical analysis was conducted and data was assessed for normality and homoscedasticity prior to analysis. In some cases Kruskal-Wallis non-parametric analysis of variance was used when data violated these assumptions and could not be corrected using a transformation. ANOVA analysis and Tukey test were also performed, and the LV_50_ were calculated by Probit analysis, using the SYSTAT statistical analysis program [37].

## 4. Conclusions

The essential oil of aerial parts of *S. ballotiflora* had insectistatic and insecticidal activities against *S. frugiperda*. β-caryophyllene and caryophyllene oxide were the main components of *S. ballotiflora* essential oil and these compounds also showed insectistatic and insecticide activities against the fall armyworm.
